# A Simple Liking Survey Captures Behaviors Associated with Weight Loss in a Worksite Program among Women at Risk of Type 2 Diabetes

**DOI:** 10.3390/nu13041338

**Published:** 2021-04-17

**Authors:** Mastaneh Sharafi, Pouran Faghri, Tania B. Huedo-Medina, Valerie B. Duffy

**Affiliations:** 1 Department of Allied Health Sciences, University of CT, Storrs, CT 06269-1101, USA; mastaneh.sh@gmail.com (M.S.); pouran.faghri@uconn.edu (P.F.); tania.huedo-medina@uconn.edu (T.B.H.-M.); 2 Department of Environmental Health Sciences & UCLA Center for Occupational and Environmental Health, UCLA Fielding School of Public Health, University of Southern California, Los Angeles, CA 90007, USA

**Keywords:** worksite health promotion, diet quality, diabetes prevention program, dietary screeners, weight loss, program evaluation, physical activity, food preference, obesity, dietary behaviors

## Abstract

In a secondary analysis, we assessed the ability of dietary and physical activity surveys to explain variability in weight loss within a worksite-adapted Diabetes Prevention Program. The program involved 58 overweight/obese female employees (average age = 46 ± 11 years SD; average body mass index = 34.7 ± 7.0 kg/m^2^ SD) of four long-term care facilities who survey-reported liking and frequency of dietary and physical activity behaviors. Data were analyzed using a latent variable approach, analysis of covariance, and nested regression analysis to predict percent weight change from baseline to intervention end at week 16 (average loss = 3.0%; range—6% gain to 17% loss), and follow-up at week 28 (average loss = 2.0%; range—8% gain to 16% loss). Using baseline responses, restrained eaters (reporting liking but low intakes of high fat/sweets) achieved greater weight loss at 28 weeks than those reporting high liking/high intake (average loss = 3.5 ± 0.9% versus 1.0 ± 0.8% S.E., respectively). Examining the dietary surveys separately, only improvements in liking for a healthy diet were associated significantly with weight loss (predicting 44% of total variance, *p* < 0.001). By contrasting liking versus intake changes, women reporting concurrent healthier diet liking and healthier intake lost the most weight (average loss = 5.4 ± 1.1% S.E.); those reporting eating healthier but not healthier diet liking (possible misreporting) gained weight (average gain = 0.3 ± 1.4% S.E.). Change in liking and frequency of physical activity were highly correlated but neither predicted weight loss independently. These pilot data support surveying dietary likes/dislikes as a useful measure to capture dietary behaviors associated with weight loss in worksite-based programs. Comparing dietary likes and intake may identify behaviors consistent (appropriate dietary restraint) or inconsistent (misreporting) with weight loss success.

## 1. Introduction

Health promotion interventions need tools to evaluate changes in diet that are feasible and useful for community settings. Dietary evaluation measures historically involve multiple dietary recalls/records and/or food frequency surveys to assess changes in energy intakes and diet quality [1]. However, intake misreporting, particularly in overweight and obese individuals [2], challenges linking of dietary changes with weight loss [3]. Novel methods of capturing diet behaviors for intervention studies are needed [2]. This paper extends our research on reported food/beverage liking as a novel and valid measure of dietary behaviors [4,5,6] that is feasible in workplace settings [7,8,9] as a potential tool for dietary evaluation.

The taste, or liking of the taste, and oral sensory properties of food are primary drivers of consumption [10]. There is growing recognition of how sensory nutrition influences health and response to dietary interventions [11]. Asking what is liked is cognitively simple and rapid to administer. Survey-reported food likes/dislikes correspond with reported intake assessed by frequency survey/food records [4,7,12,13], as well as biomarkers of consumption [4,6,14]. Food groups formed from liking surveys show internal reliability [7,8] and validity [15]. Expanding the liking survey beyond foods and beverages, including pleasurable and unpleasurable experiences, as well as physical activities, generalizes the meaning of hedonic rating scale and, through the principal of magnitude matching, supports the ability to compare ratings across individuals [16] and detect change across an intervention. Thus, could a simple liking survey add value to dietary intake measures to explain weight loss success in a workplace setting?

Improving the healthiness of a diet (i.e., diet quality) across a weight loss intervention is important for weight loss success and health promotion. In the Diabetes Prevention Program (DPP), participants who reported improvements in diet quality showed a greater level of weight loss [17] with long-term maintenance of weight loss associated with lower consumption of less healthy foods and greater consumption of healthier foods [18]. Of interest is whether diet quality indexes can be constructed from simple dietary screeners to provide insight into dietary behaviors aligned with levels of weight loss. Efforts in constructing diet quality indexes from screening food frequency questionnaires have shown promise, with reasonable association with the Healthy Eating Index (HEI) calculated from full dietary assessment methods [19]. Reliable and valid diet quality indexes can be calculated from a liking survey, associating significantly with HEI derived from full dietary assessments [4]. Liking-based diet quality indexes explain significant variability in cardiometabolic risk factors in adults [4,6,20], and associate with a level of dietary restraint, as well as with weight loss success one year after bariatric surgery [5].

Furthermore, the value of a dietary behavior method often is reported in terms of relative validity against another measure of dietary behavior. However, what if additional information on response to an intervention could be gleaned by comparing responses across multiple measures of dietary behavior? Specific to the present study, what is the relationship between reported liking and reported consumption and does this relationship inform success in a weight loss intervention? The relationship between the level of liking for a food or group of foods and the pattern of consumption can vary based on an individual’s health goals or accuracy in reporting. Liking high-fat foods but reporting consuming these foods correlates significantly with measures of dietary restraint in women in a weight loss trial [21], as an indirect measure of dietary restraint [7,21]. Appropriate levels of dietary restraint are needed to support healthy eating, weight management [22], and greater success in a weight loss intervention [21,23]. Conversely, individuals who report consuming a food but not liking the food may be trying to improve the healthiness of their diet (e.g., consuming vegetables but not reporting a high vegetable liking) or may be misreporting their dietary intake [24].

In this secondary-data analysis, we aimed to investigate the ability of liking- and frequency-based diet screeners to explain levels of weight loss in a DPP that was adapted for delivery and evaluation in a worksite. Since detecting changes in diet among women using frequency-based reporting has been challenging in DPP-based interventions [25], we hypothesized that responses from a liking-based survey for foods and beverages would show stronger associations with the level of weight loss than the frequency-based screener. Because an increase in dietary restraint has been associated with a greater level of weight loss in women [21], we tested the additional hypothesis that women who reported greater preference for, rather than intake of, high-fat sweet foods (as an indirect measure of dietary restraint), would have the greatest weight loss. Finally, as an exploratory aim, we also asked reported in liking of physical activities and sought to assess its ability to explain levels of weight loss relative to reported frequency of physical activity.

## 2. Materials and Methods

### 2.1. Participants 

This was a secondary-data analysis of a worksite-adapted DPP with a group-level randomized study (completed in 2010 [26]) and was measured at baseline, at the end of the intervention at 16 weeks (post-intervention), and at 28-weeks follow-up. All participants were employees at long-term care facilities. For the present analysis, only women were included, as they represented about 90% of original sample [26], and to avoid gender-effects on outcome reporting [25]. The inclusion criteria for the original study [26] included: ≥18 years of age, part-time/full-time long-term care employees, overweight or obese with elevated risk of diabetes [27] (Table 1). The exclusion criteria were pregnant/lactating women and those taking weight loss supplements, those who had post-bariatric surgery, as well as those with a recent ≥ 20-pound (9.1 kg) weight loss, a cancer history and/or type-1 diabetes. A database of 58 women (average age = 46 ± 11 years SD) from all levels of employment was analyzed (see Table 1). The University Institutional Review Board approved the study; all participants provided informed and written consent prior to participation. The study was conducted according to the guidelines of the Declaration of Helsinki, and approved by the Institutional Review Board of University of Connecticut—IRB Information: Protocol #: H09-129UMASSL.

### 2.2. Worksite-Adapted DPP Intervention

The intervention is previously described [26]. All participants, whether in the financial incentive arm or not, received the worksite-adapted DPP intervention. In brief, at baseline, participants completed surveys on dietary behaviors, physical activity, and barriers for achieving a healthy lifestyle. This survey information was used in an individual one-on-one consultation to help participants develop an action plan based on Small Steps Big Rewards (https://naldc.nal.usda.gov/catalog/1759336; accessed on 17 April 2021) for healthy eating and exercising. All participants signed a contract of commitment to the program, including the weight loss goal. Participants were encouraged to: (1) reduce weight by 1 to 1.5 pounds/week, achieving a modest loss (2 to 5%) from baseline; (2) reduce intake of high fat/sweet foods; (3) achieve healthy eating patterns and increase their levels of physical activity. They were also encouraged to keep a daily record of their dietary intakes and physical activities. 

Health technicians weighed and measured all participants at the beginning of the program, every week during the first four weeks, and then bi-weekly throughout the intervention, as well as at the 28-week follow-up. The weigh-in results were recorded in the participants’ weekly logs and communicated with the participants as achieving the weight loss goal or not. All participants received a weight loss manual (in lay language) with sections on self-monitoring, diet, exercise, stimulus control, social support, self-motivation, stress management, and weight management, that were discussed with strategies for problem-solving in health technician-led weekly sessions in the first four weeks and then six biweekly behavior education sessions [28]. The weigh-in logs also contained motivational messages, recipes, physical activity suggestions, and health facts.

### 2.3. Diet and Physical Activity Measures 

The diet and physical activity surveys were completed at baseline and week 16; anthropometric measures were attained at baseline, week 16 and week 28 [26]. 

The liking-based diet variables were assessed with a paper/pencil survey with a validated procedure for capturing hedonic ratings [16] and for assessing liking of food groups [8,15] and physical activities [5], as well as deriving an index of diet quality [4,6]. 

The participants were instructed to report liking/disliking for foods, physical activities, and common experiences on a horizontal bi-directional general Labeled Magnitude Scale [16], where zero was neutral, ±100 was the strongest imaginable liking/disliking, and there were intermediate labels (±1 barely, ±6 weakly, ±17 moderately, ±35 strongly, ±54 very strongly). The survey was titled, Your Likes and Dislikes, with a short paragraph describing the hedonic scale and, then, showing an example of “walking out of a dark movie on a bright sunny day” as rated between moderately and strongly disliked. Next, participants were asked to practice using the scale by rating the level of liking/disliking of “winning the lottery” and “driver cutting you off.” Having the participants start the survey by rating pleasurable and unpleasurable experiences supported the generalizability of the scale [16]. Then, they practiced with the meaning of the scale by reporting their favorite or least favorite (disliked) food and physical activity, and rated these on the scale. Finally, they rated the level of liking/disliking of the 72 items, interspersed, and in the same order for all participants. There were 57 foods/beverages (fruits, vegetables, protein, fat, sweets, salty, carbohydrates), including fitting oral sensory categories (spicy, bitter, alcoholic beverages), 8 physical activities or sedentary behaviors; and 7 were generally pleasurable (e.g., hearing your favorite music) or unpleasurable (e.g., glare of headlights) experiences. 

The liking scores were treated as a continuous variable (±100 points center rating of zero) for conceptual categorization into salty, sweet, fat, fruits/vegetables, and protein groups. Latent variable analysis identified foods loading on groups that were statistically-reliable groups (Cronbach’s alpha > 0.7). A *liking-based diet quality index* was formed from the average liking scores of these groups and a variety score [29]. The variety score was the number out of 38 nutrient-dense items (fruits, vegetables, whole grains, low-fat foods) reported as liked and numerically-converted to ±100. Similar to other dietary indexes (e.g., Healthy Eating Index [30], Diet Quality Index [31]), our previous papers [20] and guided by Dietary Guidelines [32], conceptual weights were assigned to each food group and variety score before averaging into the *liking-based diet quality index*: fruits and vegetables (+3), protein (+1), fat (−3), sweets (−3), salty food (−3), and variety (+2). To describe the study sample and consistent with previous research [29], weighed food group and variety scores ≥ 25 were assigned as optimal diet quality (weak to moderate liking for negative weighted foods and strong liking for positive weighted foods). Sub-analyses were conducted on high fat/sweet foods (cake, cake icing, cheesecake, chips, mayonnaise, whole milk, sausage). 

The *liking-based physical activity index* was comprised of 5 items (playing team sports, exercising alone, going to the gym, exercising with a partner, taking the stairs to be fit) that were averaged into a score that approached acceptable internal reliability (α = 0.68). 

The *frequency-based diet quality index* was assessed using the Insight questionnaire (J&J Health Care Systems, Piscataway, NJ, USA) [33]. Participants reported frequency, in the previous week, of consuming foods high in sweet, fats and salt, as well as whole grains, fruits and vegetables (Table 2). The number of alcoholic beverages usually consumed per week (12 oz. beer, 5 oz. wine, 1.5 oz. liquor) also was reported. For analysis, categorical responses were converted as frequency per week (e.g., 2 times per day equals 14 times per week), and treated as single variables for sweets, salty foods, whole grains, fruits, vegetables and summed for the fat foods (fried foods, red meats, cream or oil-based dressing/sauces/mayonnaise, whole milk, or cream dairy products). 

Latent variable analysis revealed two reliable factors—less healthy (sweets, fats, salty food, (α = 0.67) and healthy (whole grains, fruits, vegetables, (α = 0.75). A *frequency-based diet quality index* was the subtracted intake frequency of less healthy from healthy foods (higher index, better diet quality). An optimal index based on dietary recommendations [32] was determined to describe the study sample. Similar to reported liking, sub-analyses were conducted on the high fat/sweet foods.

**Figure 1:**
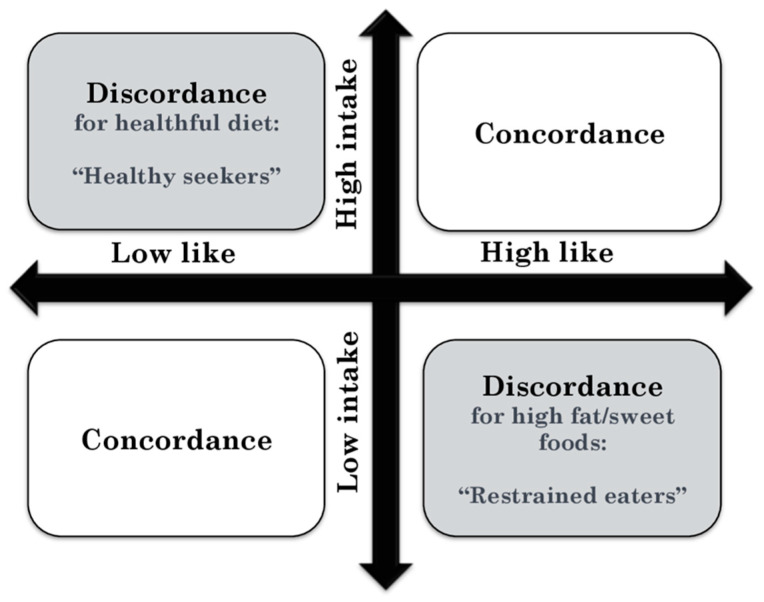
Conceptual model of agreement/disagreement in reported of liking and intake of a healthy diet (diet quality indexes) and high fat/sweet foods with high liking and low intake as an indirect measure of dietary behavior.

For evaluating relationships between reported liking versus intake, the diet quality indexes (liking-based and frequency-based) and high fat/sweet food scores (from the liking survey and frequency survey) were split at the median, forming concordant (low like/low intake, high like/high intake) and discordant (low like/high intake, high like/low intake) groups. Discordant ratings were used as an indirect measure of dietary behaviors, as shown in Figure 1. For example, participants who fell below the median value for the frequency-based diet quality index (i.e., low intake) but above the median value for the liking-based diet quality index (i.e., high-like) were labeled as “restrained eaters,” whereas participants who fell above the median value for the frequency-based diet quality index (i.e., high intake) but below the median value for the liking-based diet quality index (i.e., low-like) were labeled as “healthy seekers.” The same labels were applied to liking-based and frequency-based high fat/sweet food scores.

The *frequency-based physical activity index* was calculated from separate questions about the per week scores of mild, moderate, and vigorous physical activity by the categories of 0 days, 1–2 days, 3–4 days, and 5 or more days [33]. The specific descriptions for these physical activity categories included: mild—easy walking, bowling, mild yoga; moderate—brisk walking, slow bicycling, easy swimming; and vigorous—running, aerobics, fast bicycling. Weights were assigned to the level of exertion (mild = 1, moderate = 2, vigor = 3), multiplied by the number of days, and summed across the week to form the index for analysis.

### 2.4. Change in Weight in Response to the Intervention

Percentage in weight change from pre- to post-intervention (end of the intervention at week 16) and to the follow-up (28-week) was the primary outcome. Without shoes and excess clothing, technicians measured weight with a digital scale (Escali Digital Scale, Model BFBW200, Minneapolis, MN, USA) to the nearest ounce [26] and height to the nearest mm.

### 2.5. Data Analysis

Data were analyzed using SPSS (version 17.0; SPSS Inc, Chicago, IL, USA) and M-plus (version 7.0; Mplus Inc., Los Angeles, CA, USA). The significant criterion was *p* ≤ 0.05. Although the group membership by workplace in the incentive versus non-incentive was associated with a greater level of weight loss in the entire study of 99 participants (males and females) [26], group membership in this sub-set in the present paper did not significantly associate with the level of weight loss, the baseline diet and physical activity variables, or change in the diet and physical activity variables. The incentive variable was not a significant predictor in the testing of the study aims. Thus, the data were pooled for the analysis to address the present study aim and hypotheses.

Latent variable analysis was used to form liking-based dietary and physical activity indexes. Pearson correlation (r) analysis was used to assess the associations between liking-based and frequency-based variables. Analysis of covariance (ANCOVA) was used to test for percent weight change differences across the groups defined by concordance or discordance in liking versus intake for diet quality indexes or the fat/sweet group. Repeated ANCOVA were tested for changes in diet and physical activity variables (liking-based and frequency-based) across the intervention, by concordance versus discordance groups, and related to weight change. Variables controlled in the analysis (as appropriate) were age, education, ethnicity, and average liking ratings for common experiences as non-food standards for liking ratings [6,34]. All analyses were Bonferroni-corrected. 

Nested multiple linear regression analysis was used to identify the best model (the model that accounted for the most variation in predicting the outcome, percent weight change), from the liking- and frequency-based indexes for diet and physical activity. Consistent with a previous paper [29], we assessed two models distinguished by the order of adding the liking-based indexes to the regression analysis. The first model test added the liking-based changes in diet and physical activity after the frequency-based changes, testing its added value to predict percent weight change. The second model added the liking-based changes in diet and physical activity first, and then the frequency-based changes, testing the liking-based changes as an alternative predictor of percent weight change.

## 3. Results

### 3.1. Baseline Diet and Physical Activity

**Figure 2:**
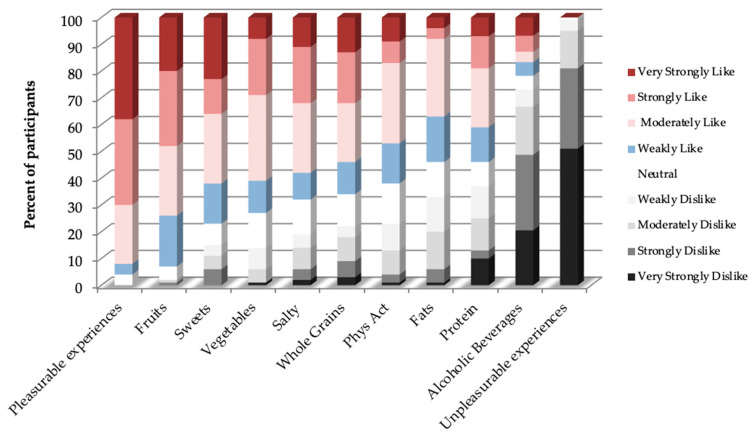
Percentage of women in each group ordered between most liked (**left**) to most disliked (**right**) groups on the liking survey at baseline (adds to 100% within a group).

The women ordered the highest/lowest liking ratings for pleasurable and unpleasurable experiences, with the most liked food group (fruits) to least liked food group (alcoholic beverages) are shown in Figure 2. 

The liking-based diet quality index showed adequate fit (χ^2^ = 225.6, df = 214, *p* = 0.28, CFI = 0.94, RMSEA < 0.05) and normal distribution (Shapiro–Wilk = 0.99, *p* = 0.87), averaging −10.8 ± 39.1 SD. Fifteen percent of the participants had high diet quality by the liking-based index. 

The women reported less frequent consumption of fat and salty foods, yet few met the recommendations of multiple daily servings of fruits, vegetables, and whole grains (Table 2). For the frequency-based diet quality index, the distribution was non-normal, positively skewed (Shapiro–Wilk = 0.86, *p* < 0.001), averaging 2.6 ± 4.4 SD. Fourteen percent of the participants had high quality diets by the frequency-based index. Consistent with the reported disliking of alcoholic beverages, 39 women said they never drank, 15 were “light drinkers” (1 to 7 drinks per week), 1 was a “moderate drinker” (8 to 14 drinks per week), and 3 did not answer. 

Reported liking and frequency relationships were strongest for alcoholic beverages and physical activity (correlations coefficients ranged in the 0.4′s, *p* < 0.001). Associations were lower for the other food groups and diet quality indexes. The correlations coefficients ranged in the 0.3’s, *p* < 0.05 for sweets, salts, fats, whole grains and overall diet quality, as well as in the 0.2′s (*p* < 0.2) for fruits and vegetables. Concordance and discordance groups were formed for agreement versus disagreement for liking versus intake of high fat/sweet foods, and overall diet quality. For high fat/sweet foods in the sub-analysis, 34 (59%) participants were concordant in reported liking/intake, 24 (41%), reported high like/low intake (*n* = 14), or low like/high intake (*n* = 10). The groups were nearly an equal percentage (~25%) for the concordance groups for agreement versus disagreement for liking versus intake for the diet-quality indexes.

For physical activity liking, 12.5% of women reported more than strong liking for physical activity by the liking-based physical activity index. This index had an adequate fit (χ^2^ = 8.5, df = 7, *p* = 0.30, CFI = 0.98, RMSEA < 0.05). For physical activity frequency, and consistent with report physical activity liking, only 12.5% of women met the recommendation of 150 min of moderate or 75 min of vigorous physical activity per week [35].

### 3.2. Primary Outcome of Weight Loss 

By post-intervention, participants averaged 3% weight loss, consistent with the program’s goals. Weight change ranged from 6% weight gain to 17% weight loss; >85% lost weight by post-intervention. By the 28-week follow-up, average weight loss fell to 2% (ranged from 8% weight gain to 16% weight loss). Although some women gained weight, approximately 50% sustained the weight loss or lost more weight. There was no significant association between weight change and age, ancestry, or education.

At the baseline, diet quality or physical activity, assessed by single method (from liking or frequency surveys), were not associated with significant differences in weight loss. However, following Figure 1, differences in weight loss were explained by examining participants who were discordant and concordant in liking versus frequency measures. At post-intervention (week 16), participants who were discordant in liking-based versus frequency-based for the diet quality indexes (either high like/low consumption or low like/high consumption) lost significantly more weight by post-intervention than those who were concordant (4.1 ± 0.9% versus 1.7 ± 0.8 S.E., respectively; *F* = 4.1, *p* = 0.05). Furthermore, at post-intervention, levels of weight loss were different for participants who were discordant for liking and consumption of high fat/sweet foods (*F* = 7.7, *p* < 0.01); those who reported high liking but low consumption (i.e., restrained eaters) lost more weight than those who reported high liking and high consumption (4.7 ± 1.1% versus 1.4 ± 1.1 % S.E., respectively; *p* < 0.05) of these foods. At the 28-week follow-up, the discordant group for high fat/sweet foods (high liking but low consumption) maintained higher weight loss than the concordant (high liking and high consumption) group (3.5 ± 0.9% versus 1.0 ± 0.8% S.E., respectively; *F* = 4.4, *p* < 0.05). 

### 3.3. Changes in Reported Diet and Physical Activity 

As shown in Table 3, the liking-based diet quality index improved significantly by 118% from pre- to post-intervention. At post-intervention, 20% of participants met the optimal criteria. Although the ranking of most-to-least liked foods at post-intervention was similar to that at baseline (Figure 2), the sub-analysis showed the liking changed primarily for high fat/sweet foods (42% decrease). As expected, there were no significant changes from baseline to post-intervention for reported liking of the pleasurable (36.3 ± 4.0 versus 33.4 ± 4.1 S.E., *p* = 0.6) or the unpleasurable (−65.2 ± 2.5 versus −60.0 ± 3.5 S.E., *p* = 0.14) items.

The frequency-based diet quality also improved by 148% (Table 3); approximately half of participants reported achieving optimal levels at post-intervention. Consumption of individual food groups also significantly increased, except for whole grains and alcohol. However, the non-normal distribution of all frequency-based data required logarithmic transformation to approximate normality. The transformed data indicated weaker improvement; changes in consumption of fats became non-significant. Overall, at post-intervention, the women continued to report lower consumption of high fat/sweet/salty foods, yet increased consumption of fruits, vegetables, and whole grains.

The next analysis examined whether concordance or discordance in liking versus intake at baseline was associated with differences in change in liking across the intervention using a mixed-model repeated measure ANCOVA. For the liking versus frequency-based diet quality indexes, there were no significant differences in liking change between participants who reported discordance in liking versus intake at the baseline. However, high fat/sweet foods showed significance within (*F* = 5.5, *p* < 0.05) and between (*F* = 5.8, *p* < 0.01) effects. Participants who reported liking but not eating high fat/sweet foods (i.e., restraint eaters) reported significant reductions in liking of high fat/sweet foods (*p* < 0.05), whereas participants who reported concordance between liking and intake (low/low, high/high) reported non-significant reductions. Finally, participants who were low liking/high intake at baseline reported non-significant increases in liking for high fat/sweet foods.

The strength of the associations between reported liking and intake also changed from pre- to post-intervention. Pearson’s coefficients became stronger for sweets, vegetables, and fruits, ranging in the 0.4′s (*p* < 0.01)—still significant but weaker for salty foods (r = 0.33, *p* < 0.05), and only trending for diet quality (r = 0.3, *p* = 0.06). For physical activity, the associations between liking and frequency-based indexes remained significant (r = 0.36, *p* < 0.05). By post-intervention, improvements in physical activity by liking and frequency were highly correlated (r = 0.48, *p* = 0.003), yet only the frequency-based physical activity showed significant improvements from baseline to post-intervention at 16 weeks (Table 3).

### 3.4. Associations between Weight Loss Success and Changes in Diet Quality and Physical Activity Indexes

Two nested regression models tested liking as added value (model 1) and alternative (model 2) predictors of weight loss at post-intervention (Table 4). In model 1, changes in the frequency-based diet quality index alone had an insignificant predictive ability to explain weight change (6%, *p* = 0.42). However, the addition of the liking-based diet quality index contributed 46% (R^2^, *p* < 0.001) more to the total variance explained (i.e., liking as a significant value-added predictor). In model 2, the liking-based diet quality index alone explained 44% of total variance, significantly predicting weight change (*p* < 0.001) as an alternative predictor. Adding the frequency-based diet quality index failed to add significant predictive ability. Neither model showed significant effects of changes in physical activity frequency or liking on percent weight change from pre- to post-intervention.

Similar results were found by the follow-up; improvement in the liking-based diet quality index was a significant value added and alternative predictor (for both, R^2^ = 37%, *p* = 0.001) of weight change. Consistent to post-intervention, improvement in liking-based diet quality index was the sole significant predictor of weight loss at follow-up (β = 0.52, *p* < 0.01). Models tested with the covariates together explained less than 5% of variability in weight change, with no influence on the results presented in Table 4.

Although the women reported significant improvements in diet quality from pre- to post-intervention on the frequency screener (Table 3), these gains were not significantly associated with the weight change (Table 4). Furthermore, since the correlation between change in the liking- and frequency-based diet quality index was non-significant (*p* = 0.50), concordant/discordant groups were formed from median splits. ANCOVA revealed significant differences in average weight change between the concordant/discordant groups from baseline to post-intervention (*F* = 3.9, *p* < 0.05). Women who reported concordant improvements in liking- and frequency-based diet quality averaged the highest weight loss (5.4 ± 1.1% S.E.) versus those who reported improvements in frequency but not liking averaged gain of weight (0.3 ± 1.4% S.E., *p* < 0.05). The latter group may represent misreporters. Further exploration of these misreporters (i.e., reported improvements in frequency- but not in liking-based diet quality indexes) revealed greater intakes of all healthy foods, significant for fruits (*F* = 15.9, *p* < 0.01) and trending for the other groups (*p* < 0.10), and lower intakes of all less healthy foods, significant for red meats (*F* = 10.5, *p* = 0.01), oily foods (*F* = 5.3, *p* = 0.05), and trending for processed meat (*F* = 4.1, *p* = 0.08). 

## 4. Discussion

Community-based evaluation studies and trials need dietary methods and tools that are feasible and able to detect changes in behaviors associated with primary outcomes. The present study, from a secondary analysis, examined levels of weight loss in women explained by liking- and frequency-based dietary screeners in response to a worksite-adapted DPP at 16-week post intervention and 28-week follow-up. Baseline responses to the liking survey, combined with the food frequency screener, illuminated differences in the percentage of weight loss. Participants who reported a high liking for high-fat/sweet foods but a low intake of these foods (i.e., restrained eaters) at baseline reported significant reductions in liking of these foods across the intervention, and lost a significantly greater percentage of weight at 16-week post-intervention and 28-week follow-up. While the participants reported significant improvements in diet quality across the intervention on the frequency screener (increases in healthy foods and reductions in less healthy foods), only improvements in the liking-based index explained significant variations in percent weight loss at post-intervention and follow-up (decreased liking of less healthy, high-fat, sweet foods). At post-intervention, the greatest weight loss was observed among participants who reported improvements in diet quality by the liking and frequency screeners. Finally, although physical activity liking and frequency were significantly correlated, neither physical activity reports at baseline nor change with the intervention explained significant variability in the percentage of weight loss.

We support our hypothesis that the liking survey, particularly the assessment of food and beverage liking, would add to the ability to explain levels of weight loss in response to a worksite-based intervention. Dietary assessment tools used in experimental settings may fail in community-based settings, in part, because of participant burden and misreporting [2,3]. Our findings join others [21,36,37] to support liking surveys as dietary evaluation tools. Liking or preference falls under the broad umbrella of behaviors that lead up to what is consumed [38], with liking of the taste or sensory components of foods and beverages as a primary driver of consumption [10]. Survey-reported liking appears to be a proxy of dietary intake, correlating with reported intake in this study and others [4,7,12,13], as well as biomarkers of consumption [4,6,14]. Consistent with previous studies [4,6,20] and our latent variable analysis, the diet quality index formed from the liking survey had internal consistency (reliable), normal distribution, and construct validity (match between observed and expected internal structure). The food frequency-based scores required transformation to approximate normality before parametric statistical analysis. 

Previous research has supported that brief dietary screeners could detect changes in responses to community-based weight loss intervention, but more work was needed [39] to capture change in fat consumption and dietary quality [40]. In the present study, there were improvements in the liking-based diet quality index, yet the findings were driven by reduction in the liking for high fat/sweet foods. These findings agree with decreases in fat and sweet preference with weight loss [41], including after recovery from bariatric surgery [42,43,44,45,46,47] with the level of weight loss [5,48] and improvements in cardiometabolic risk [49]. However, healthy diets must also include increases in adequacy or healthy components. According to a critical assessment, weight loss success and long-term weight control result from greater consumption of vegetables and fruits [50] and whole grains [51], which support satiety through textural complexity, the need to chew, and slowing down rates of eating [52].

Furthermore, improvements in diet quality from a frequency screener failed to associate with greater weight loss unless coupled with improvements in healthy food liking. Women who only reported improved consumption but not liking of healthy foods averaged one pound weight gain by post-intervention; those who reported improved liking and consumption averaged an eight-pound weight loss. Non-dietary factors, such as social desirability, may lead to over-reporting of diet healthfulness [53]. Liking-based dietary reports may be less prone to misreporting bias for the following reasons: (1) individuals may perceive that reporting liking is less likely to raise social judgment; (2) recalling likes/dislikes requires only affective memory [54] compared to intake reports, which require factual memory; (3) non-foods serve as standards of comparison; and (4) liking reports showed predictive ability in the direction expected (i.e., greater improvement associated with greater weight loss). 

Dietary restraint in the current study was assessed indirectly by discordance in reported liking versus intake, as previously addressed [7,8,55], and linked to weight loss success [21]. The distribution of liking ratings in the current study (Figure 2) is similar to those reported by individuals with morbid obesity contemplating bariatric surgery [56], as well as young adults (including those with depression/anxiety) [6]. Liking high-fat foods but reporting consuming these foods correlates significantly with dietary restraint, measured by the Eating Inventory [57], as shown in women from a laboratory-based study, a cross-sectional analysis of a free-living sample, and a six-month weight loss trial [21]. The present study extends previous findings, showing that indirectly assessed dietary restraint was associated with weight loss by follow-up, and was consistent with dietary restraint assessed directly (Three-Factor Eating Questionnaire [58,59], Dutch Eating Behavior Questionnaire [60]). Dietary behaviors characterized by cognitive control of eating, as well as less disinhibited and emotional eating, support long-term weight success [60,61,62,63]. An indirect assessment of dietary restraint may have less reporting bias than directly asking participants about dietary restraint behaviors. 

Liking for and reported frequency of physical activity were correlated at baseline and post-intervention, but there was significant change only for reported physical activity from baseline to post-intervention. However, neither a change in frequency-based nor in liking-based physical activities indexes explained significant variance in the level of weight loss across the intervention. According to a critical review of the literature, weight loss occurs with physical activity, accompanied with restricted energy intake, and obtaining a sufficient amount of physical activity (e.g., 150 min/week of moderate to intense physical activity), including brisk walking [64]. The women in the present study may not have had achieved enough improvements in physical activity to reach the moderate to intense physical activity level. A study with the full dataset from the worksite-modified DPP found that reported the level of physical activity at post-intervention (at levels from mild to vigorous) explained levels of weight change through exercise self-efficacy or the confidence the participant (both women and men) felt for having the motivation to exercise for six months or longer [65]. The present analysis of a sub-sample of only women from the original study did not consider self-efficacy. However, that there was less improvement in liking than frequency of physical activity may have indicated that the women were participating in activities that really were not preferred. Previous research supports that exercises have to be liked to be sustainable [66]. Thus, asking about physical activity may help to identify the most preferred activities across health promotion programs. Comparing the liking of physical activity with a frequency assessment could identify ways to support enjoyable and sustainable physical activity behaviors.

This study has limitations. Despite the findings that the dietary screeners, particularly the liking survey, explained success in weight loss, the results should be assessed with full measures of dietary intake [1], as well as physical activity [67]. In addition, the small sample size of only women reduces the generalizability of the findings. Nonetheless, the study sample of women was ideal for an intervention to improve dietary and physical activity, with the majority having elevated weights, risk of diabetes, and less healthy diet and physical activity behaviors. The group averaged a level of weight loss across 16 weeks similar to other DPP trials (e.g., [68]). Consistent with the present study, adults with chronic disease risk factors have poorer quality diets [69]. Employee-based interventions to improve the health of workers in high-stress work environments, such as long-term care, are highly needed, coupled with safety and fully integrated into the workplace [70].

## 5. Conclusions

Community-based weight loss programs need feasible tools for evaluating changes in dietary behavior that are associated with levels of weight loss. The present study supports that responses to a simple survey of food likes/dislikes explained levels of weight loss at post-intervention (16 weeks) and follow-up (28 weeks) in women who participated in a worksite-based adaptation of the Diabetes Prevention Trial. Greater weight loss was associated with reported improvements in the liking-based diet quality index, primarily through reductions in liking of high-fat, sweet foods. There was value in comparing liking responses to frequency screener responses. Women who reported concurrent improvements in liking and eating a healthier diet lost the most weight. Discordance frequency to liking responses can serve as an indirect measure of dietary restraint (reporting lower intake of more liked fat/sweet foods) or misreporting (reporting eating healthier but not liking healthier diets). Future studies are needed to replicate these findings against more precise dietary intake measures, especially in research-related obesity interventions.

## Figures and Tables

**Table 1:** Demographic and health variables of female employees from four long-term care facilities (*n* = 58).

	%	Mean (SD)
Age		46.0 (11)
20 to <30 years	5	
≥30 to 40 years	20	
40 to 50 years	36	
50 to 60 years	25	
≥60 years	14	
Ancestry		
White	46	
Black	45	
Other	9	
Education		
<High School	7	
High School	44	
College/grad school	49	
BMI (from measured weight/height)		34.7 (7.0)
Overweight (25 ≤ BMI < 30)	22	
Obese I (≤30 BMI < 35)	40	
Obese II (35 ≤ BMI < 40)	17	
Obese III (BMI ≥ 40)	21	
Measured waist circumference (inches)		41.2 (5.0)
Normal (<35 inches)	10	
Elevated risk (≥35 inches)	90	
Measured blood pressure (mm Hg) †		
Normal (<120/80)	40	
Pre-hypertension (120/80–139/89)	40	
Hypertension (≥140/90)	20	
Reported hypertension		
No	68	
Yes	32	
Reported elevated cholesterol		
No	67	
Yes	33	
Reported type 2 diabetes		
No	85	
Yes	16	
Diabetes risk ††		
No risk (0–5)	0	
Low risk (6–8)	9	
Moderate risk (9–12)	63	
High risk (13–20)	28	

† categories of blood pressure by Centers of Disease Control criteria https://www.cdc.gov/bloodpressure/facts.htm (accessed on 17 April 2021). †† scores from diabetes screener [27] defined the diabetes risk categories.

**Table 2:** Percentage of women who fell in frequency categories from a food frequency screener at baseline in a modified DPP.

Food Group †	Never-1x/wk	1–4x/wk	5–7x/wk	2x/day	≥3x/day
Fried foods	53	34	11	2	0
Red meat (steak, hamburger, pork, or lamb)	32	57	9	2	0
Processed meat (hot dogs, bologna, salami, pepperoni, sausage, or bacon)	53	40	7	0	0
Cream- or oil-based salad dressing, cream sauces, mayonnaise	52	36	12	0	0
Whole milk dairy products (whole milk, yogurt, ice-cream, cheese, or butter)	29	52	13	3	3
Cookies, pastries, cakes, or chocolate candy	34	46	16	2	2
Whole grain cereals, breads, pasta	7	45	43	5	0
Fresh, frozen, or canned fruits or fruit juices	32	32	18	11	7
Fresh, frozen, or canned vegetables or vegetable juices	20	25	34	18	3

† Frequency categories within a food group added to 100%.

**Table 3:** Average (±S.E.) absolute values for the diet and physical activity variables at baseline and post-intervention.

	Liking-Based Measures ^a^			Frequency-Based Measures ^a,b^		
	Baseline	Post-Intervention	*F*, *p* Values †	Baseline	Post-Intervention	*F*, *p* Values †
High fat/sweet foods	19.5 ± 3.6	11.5 ± 4.7	10.7, *p* < 0.01	15.3 ± 1.8	11.2 ± 1.2	5.0, *p* < 0.05
Fat ^c^	13.6 ± 4.1	4.5 ± 4.7	6.0, *p* < 0.05	11.2 ± 1.4	8.5 ± 1.0	3.1, *p* = 0.09
Sweets	22.6 ± 4.6	16.9 ± 5.5	6.9, *p* = 0.01	3.2 ± 0.6	2.5 ± 0.4	3.4, *p* = 0.07
Salty	13.5 ± 4.2	5.8 ± 4.6	4.7, *p* < 0.05	2.3 ± 0.2	1.8 ± 0.2	7.6, *p* < 0.01
Alcohol	−30.5 ± 7.0	−36.0 ± 4.6	1.1, *p* = 0.3	1.3 ± 0.1	1.2 ±0.1	2.0, *p* = 0.17
Fruits	37.0 ± 3.4	37.7 ± 3.0	0.1, *p* = 0.9	4.9 ± 0.8	9.1 ± 1.2	12.1, *p* = 0.001
Vegetables	18.5 ± 4.7	23.4 ± 4.5	2.4, *p* = 0.12	6.1 ± 0.8	9.4 ± 1.3	6.0, *p* < 0.05
Whole Grains	20.3 ± 5.5	23.0 ± 4.6	0.24, *p* = 0.63	5.1 ± 0.5	6.5 ± 0.9	1.8, *p* = 0.19
Diet Quality Index	−9.3 ± 7.0	1.7 ± 10.1	6.5, *p* = 0.01	2.5 ± 0.7	6.1 ± 1.0	4.8, *p* < 0.05
Physical Activity Index	18.4 ± 1.0	19.4 ± 2.4	0.3, *p* = 0.6	27.3 ± 2.6	36.1 ± 3.9	7.8, *p* < 0.01

† Repeated measures ANCOVA assessing changes from baseline to post-intervention (16 weeks) with Bonferroni adjustment. ^a^ Food group values on the liking scale (±1 barely, ±6 weakly, ±17 moderately, ±35 strongly, ±54 very strongly) and frequency scale (weekly consumption). ^b^ Frequency variables were not normally distributed (except for physical activity score), and thus logarithmically transformed for repeated measures ANCOVA. ^c^ Fat intake is the sum of frequency for four high-fat food groups (fried foods, high-fat proteins, oily foods, and high-fat dairy provided in the frequency screener.

**Table 4:** Relative contributions of changes in liking- and frequency-based indexes on percent weight change from pre- to post-intervention in women using nested regression analysis.

			Improvement in R^2^		Standardized Beta, *p*	
Step	Predictor	Total R^2^		*p* Change	Set 1 ^a^	Set 2 ^b^
**Model 1–liking as added value predictor**						
1. Frequency added first		6%		*p* = 0.42		
	Changes in diet				0.08, *p* = 0.68	0.18, *p* = 0.21
	Changes in PA				0.21, *p* = 0.27	0.22, *p* = 0.21
2. Liking added second		52%	46%	*p* < 0.001		
	Changes in diet					0.60, *p* < 0.001
	Changes in PA					0.24, *p* = 0.16
**Model 2–liking as alternative predictor**						
1. Liking added first		44%		*p* < 0.001		
	Changes in diet				0.63, *p* < 0.001	0.60, *p* = 0.001
	Changes in PA				0.11, *p* = 0.44	0.24, *p* = 0.16
2. Frequency added second		53%	9%	*p* = 0.11		
	Changes in diet					0.18, *p* < 0.21
	Changes in PA					0.22, *p* = 0.21

^a^ Set 1 includes standardized beta and *p* values for variables used in the first step of nested regression analysis. ^b^ Set 2 includes standardized beta and *p* values when new predictors were added to the final step of nested regression analysis.
